# Cortical and white matter mapping in the visual system-more than meets the eye: on the importance of functional imaging to understand visual system pathologies

**DOI:** 10.3389/fnint.2014.00068

**Published:** 2014-08-27

**Authors:** Noa Raz, Netta Levin

**Affiliations:** fMRI Unit, Neurology Department, Hadassah Hebrew University Medical CenterJerusalem, Israelm

**Keywords:** visual cortex, visual pathways, visual pathologies, functional MRI (fMRI), Diffusion Tensor Imaging (DTI)

## Abstract

Information transmission within the visual system is highly organized with the ultimate goal of accomplishing higher-order, complex visuo-spatial and object identity processing. Perception is dependent on the intactness of the entire system and damage at each stage—in the eye itself, the visual pathways, or within cortical processing—might result in perception disturbance. Herein we will review several examples of lesions along the visual system, from the retina, via the optic nerve and chiasm and through the occipital cortex. We will address their clinical manifestation and their cortical substrate. The latter will be studied via functional magnetic resonance imaging (fMRI) and Diffusion Tensor Imaging (DTI), enabling cortical, and white matter mapping of the human brain. In contrast to traditional signal recording, these procedures enable simultaneous evaluation of the entire brain network engaged when subjects undertake a particular task or evaluate the entirety of associated white matter pathways. These examples provided will highlight the importance of using advanced imaging methods to better understand visual pathologies. We will argue that clinical manifestation cannot always be explained solely by structural damage and a functional view is required to understand the clinical symptom. In such cases we recommend using advanced imaging methods to better understand the neurological basis of visual phenomena.

In order for visual perception to occur, the physiological signal initiated by the photoreceptors in the retina must travel all the way to the visual cortex at the back of our brain. This long journey requires a set of long-range white matter tracts to communicate the signals.

The retinal ganglion cell axons leave the eye by way of the **optic nerve**, and there is a partial crossing of axons at the optic chiasm. After the chiasm, the axons (which now carry binocular information) are referred to as the **optic tract**. The optic tract wraps around the midbrain to get to the Lateral Geniculate Nucleus (LGN), where all the axons synapse. From there, the LGN axons fan out through the deep white matter of the brain as **optic radiations**, which will ultimately travel to the primary visual cortex. The **occipital-callosal fiber tract** that connects the two occipital lobes via the corpus callosum is a key pathway for the flow of information between the two hemispheres (Barr and Kiernan, 1993). The visual information is then processed in the visual cortex. The visual cortex is divided hierarchically into primary (lower in the hierarchy) areas located in the occipital lobe and late (higher in the hierarchy) areas, which are located in the temporal and parietal lobes. Mapping in primary visual areas is sensitive to the stimulus' topography, known as **retinotopic mapping**; adjacent points in the visual field are represented close to each other in the visual cortex such that the cortical representation reflects the retinal geometry (Wandell, 1999). The upper visual field is mapped within the lower lip of the calcarine sulcus and the lower visual field in its upper part; similarly, the right visual field is mapped in the left hemisphere and the left visual field in the right side. The eccentricity axis along the central to peripheral visual field is represented topographically along the calcarine sulcus from the posterior to the anterior parts. A relatively large cortical area is devoted to the fovea; large numbers of small receptive fields' neurons process information from a small region of the visual field, enabling the fine spatial resolution that characterizes foveal vision.

Mapping in higher visual areas (higher in the hierarchy) located in the occipito-parietal and occipito-temporal regions is less sensitive to stimuli topography and more involved in processing the **functional aspects** of the stimuli. This includes areas in the ventral and the dorsal streams that analyze an object's identity and object's usage, respectively (Goodale and Milner, 1992).

The possibility of mapping the functioning human visual cortex has developed during the last two decades using Functional MRI (fMRI) which utilizes the neuro-vascular coupling principle: in response to a visual task, neural activity in a specific cortical visual region will trigger change in the regional blood flow which will be captured in the MRI scan (Logothetis, 2008). *Retinotopic mapping* is obtained by presenting simple visual stimuli to the different visual fields' portions. For example, in order to map the eccentricity axis from the central to the peripheral visual field, an expanding ring stimulus is used. The subject views an expanding flashing checkerboard circular stimulus that gradually expands from the center to the periphery of the visual field. This stimulus creates a wave of neuronal activity, which moves along the calcarine sulcus from the posterior to the anterior region (Engel et al., 1997). An example of such mapping can be seen in Figure 1. The cortical magnification factor should be noted: a large cortical area is devoted to the processing of the 2° of the central visual field.

**Figure 1:**
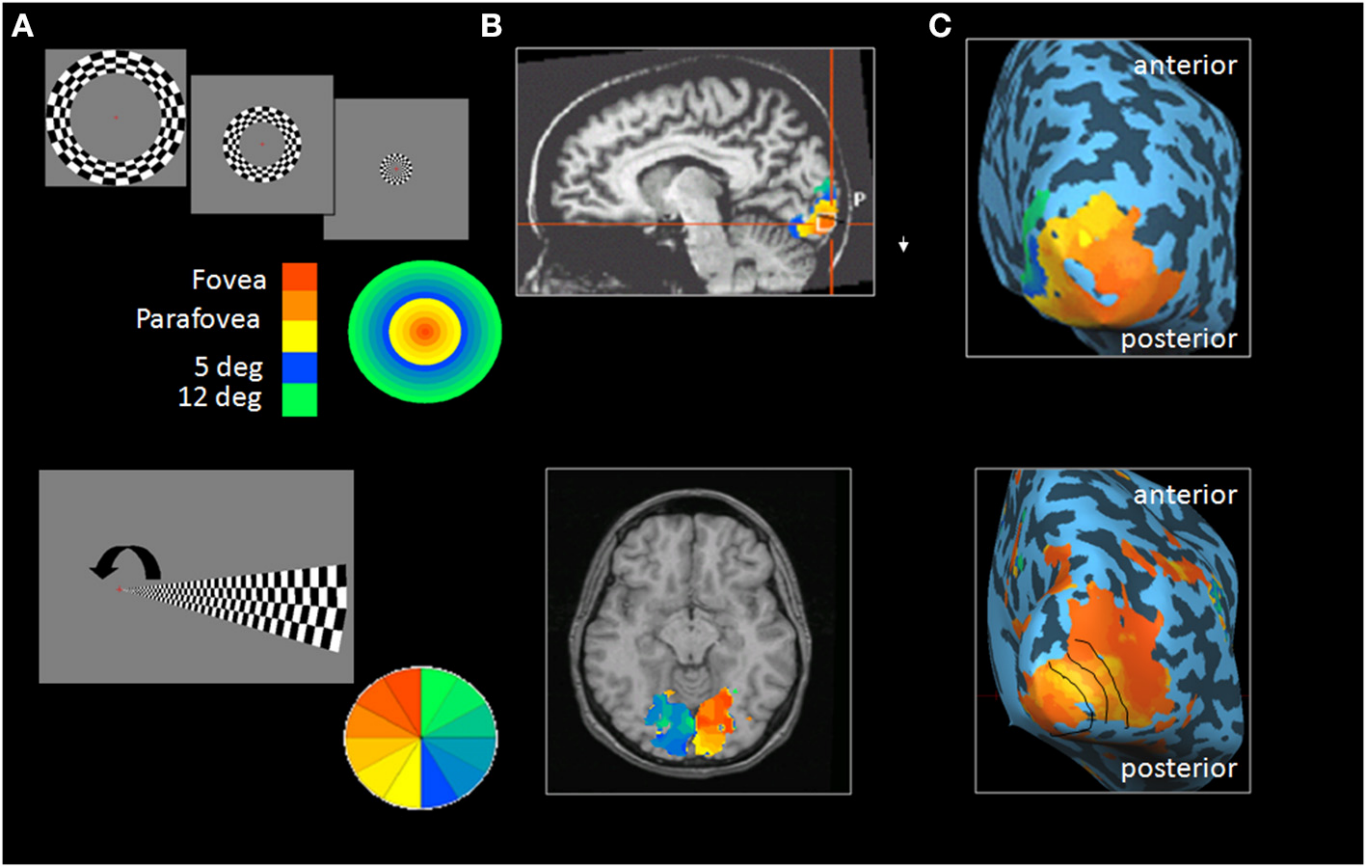
**Retinotopic mapping in early visual areas. (A)** Top: The expanding ring stimulus used to map the visual field eccentricity axis from center to periphery. Bottom: The rotating wedge stimulus used to differentiate between early retinotopic regions. Color scales indicate the correspondence between the position of stimuli within the visual field and cortical maps. The cortical activity pattern in response to the expanding ring (top) or the rotating wedge (bottom) is overlaid on T1 slices (presented in neurological convention, i.e., right in the picture correspond to right hemisphere), **(B)** and on the inflated posterior view of the right hemisphere **(C)**. The arrow represents the wedge rotation.

*Functional mapping* is obtained using more complex visual stimuli; targeting the cortical areas activated during the presence, as compared with the absence, of specific visual attributes. For example in order to map cortical areas specialized in object recognition, the subject is alternately presented with either pictures of objects or a scrambled version of these objects (Malach et al., 1995). While the basic parameters of the stimuli are identical in both conditions, object perception exists in the first but not in the second condition. Likewise, in order to map cortical areas that specialize in motion processing, the subject will be presented with moving and stationary objects identical in all aspects except their mobility (Tootell et al., 1995). Figure 2 demonstrates the lateral occipital cortex (LOC), specifically activated during visual object processing and the motion sensitive region, MT.

**Figure 2:**
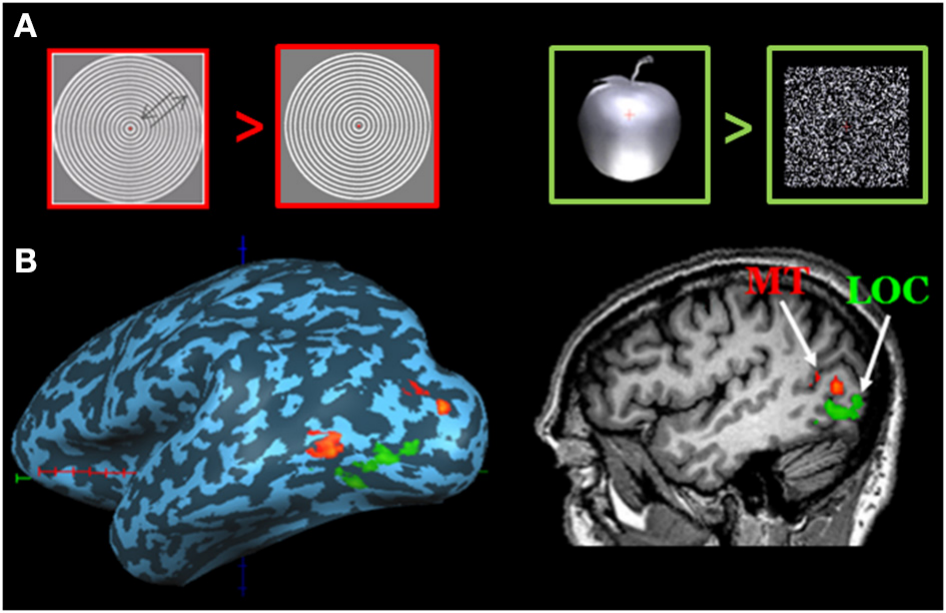
**Mapping motion and objects selective areas. (A)** Stimuli used for mapping motion-related areas (left) and object-related areas (right). The orange plus on the apple represents fixation cross. **(B)** MT region (red) was defined as a region in the posterior banks of the inferotemporal sulcus that responds more strongly to moving vs. stationary low contrast grating. LOC (green) was identified by having grater activation during object than scrambled object stimuli. The cortical activity is overlaid on sagital view and on the inflated lateral view of the left hemisphere. Optic tracts delineation in the patient (blue fibers), superimposed on axial and coronal view.

Early usage of fMRI was dedicated to mapping the healthy human visual cortex, adding a significant layer to the data collected in animal and human lesion studies. The ability of the fMRI signal to reflect neuronal mass activity (reflecting the average activity of ~ millions of neurons) served to explain the functioning of distributed systems, such as our visual pathways. This accumulated knowledge of the intact visual pathways provided the basis for understanding the neuronal substrate of unexplained visual dysfunctions, which cannot be addressed via standard anatomical imaging regime. fMRI has contributed, for example, to our understanding of the phenomenon of blindsight, in which a patient with cortical damage resulting in hemianopia retains some residual abilities within his blind field. fMRI studies in these patients demonstrated powerful activation in area MT, despite nearly complete destruction of the primary visual region (V1) (ffytche et al., 1996; Goebel et al., 2001). This provided powerful support for the hypothesis that blindsight is based on signals that travel via alternate pathways, projecting via the superior colliculus and pulvinar to area MT (Wandell and Wade, 2003).

Similarly, fMRI was useful for explaining visual hallucinations, demonstrating that hallucinations of color, faces, textures, and objects were accompanied by signal increases in visual areas whose specializations matched the induced hallucination contents (Ffytche et al., 1998) and by changes in fMRI connectivity between LGN and the visual cortex (Ffytche, 2008). fMRI has also been useful for explaining the neuronal basis of compensatory behavior in patients with no visual inputs, recruiting the visual cortex to improve non-visual functions. As was demonstrated in congenital blindness, lower, and higher-order cortical visual regions are activated during non-visual tasks. Furthermore, these activations were associated with improvement of non-visual functions (e.g., Sadato et al., 1996; Amedi et al., 2003; Raz et al., 2005).

Tracking white matter is done using Diffusion Tensor Imaging (DTI). The method is based on diffusion, the random motion of water molecules in the extra- and intra-cellular spaces. When the motion of molecules is limited by *tubular* structures such as the axons, diffusion will be mainly along the direction of the axonal bundle, rather than in the perpendicular plane (anisotropic diffusion). Using this method, information regarding localization and directionality of the fibers as well as evaluation of the quality of their cohesiveness and their ability to conduct the neuronal signal can be obtained (Basser et al., 1994; Mori et al., 1999). Figure 3 demonstrates reconstruction of the visual pathways using DTI and fiber tractography, and shows the *optic tract* that connects the optic chiasm to the LGN, creating a rather thin and compact fiber bundle; and the *optic radiation* that connects the LGN to the occipital cortex containing its anterior segment, the Meyer's loop.

**Figure 3:**
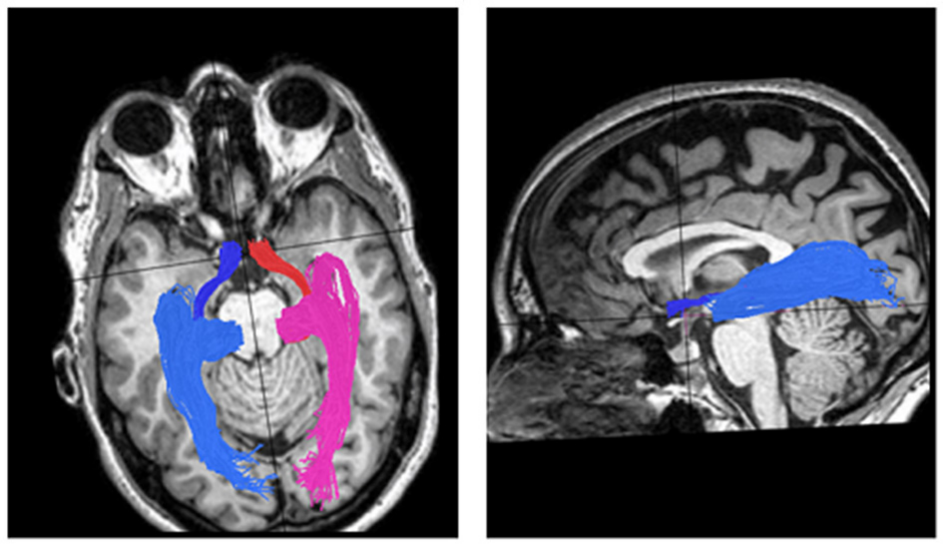
**DTI visual pathways fiber groups superimposed on T1 images. Left:** axial view of the optic tracts and radiations in one control subject. The optic tracts, which connect the optic chiasm to the LGN, are colored red for the right side and blue for the left side. The right and left optic radiations, which connect the LGN to the calcarine cortex, are colored pink for right and light blue for left. **Right:** sagital view of the left optic tract and radiation.

Fibers' cohesiveness may be evaluated by four diffusion properties: mean diffusivity (MD); fractional anisotropy (FA, a scalar value describing the degree of anisotropy of a diffusion process); diffusion parallel to the principal fiber direction (axial diffusivity, AD); and diffusion perpendicular to the principal fiber direction (radial diffusivity, RD) (Basser et al., 1994). Animal model studies of axon and myelin pathology have demonstrated that reduced AD is associated with axonal pathology, whereas increased RD is associated with demyelination (Song et al., 2003).

Visual perception is dependent on functioning of the visual system in its entirety and perception may be impaired by damage to any of the pathway's components, starting at the eye itself, through the visual pathways and continuing to the visual processing site, the cortex. Herein we will briefly review examples of different insults along the visual system that we have encountered in patients who have presented during recent years, and their clinical and imaging manifestations (Figure 4).

**Figure 4:**
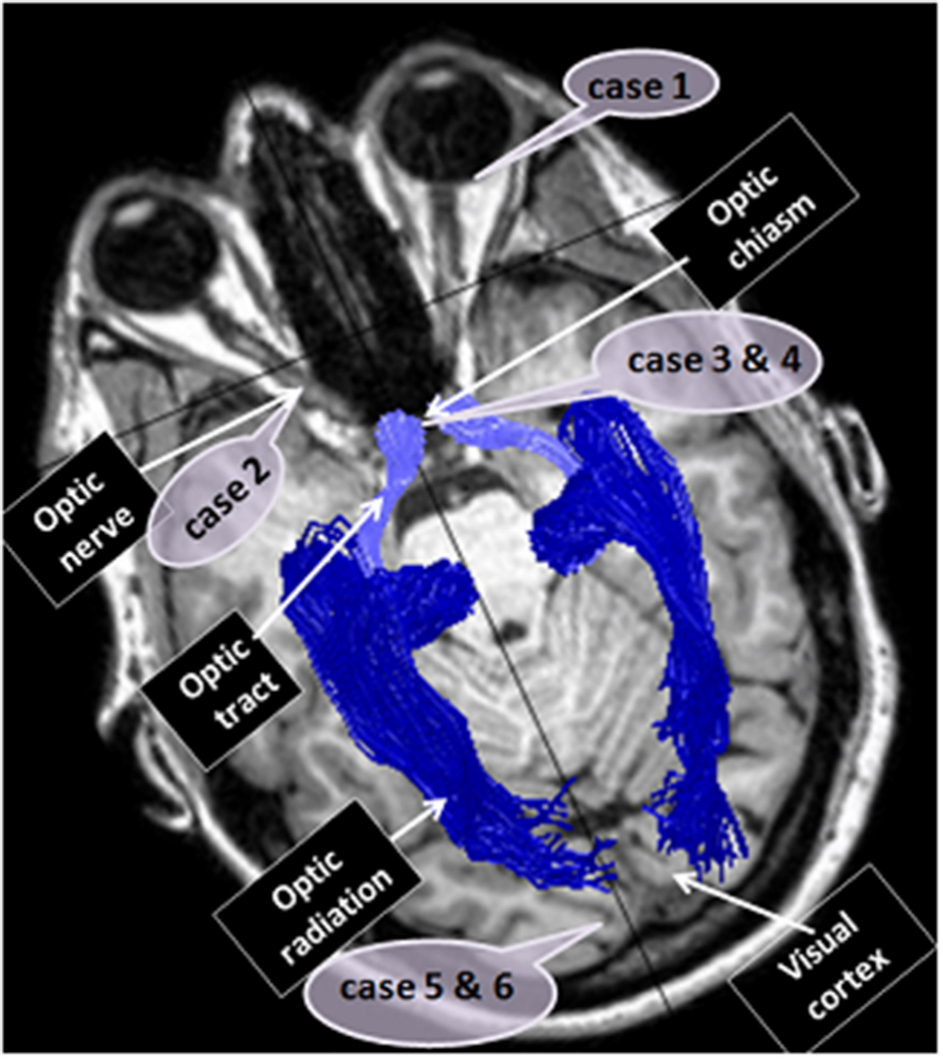
**Case reports localizations**. The figure localizes the sites of dysfunction in the different cases reported: case 1—the eye, case 2—the optic nerve, cases 3 and 4—optic chiasm, cases 5 and 6—the visual cortex.

## Acquired eye insult in the young (figure 5)

Case 1: MM, a 53-year-old male, lost his left eye and became blind in the right due to corneal damage at the age of 3. At age 46, following more than 40 years of blindness, MM underwent corneal and limbal stem-cell transplant in his right eye and regained his right retinal image. Nevertheless, even 7 years following surgery, his visual abilities remained severely limited, and he didn't rely on vision for his daily life functioning (poor spatial resolution, especially in high frequencies were evident).

**Figure 5:**
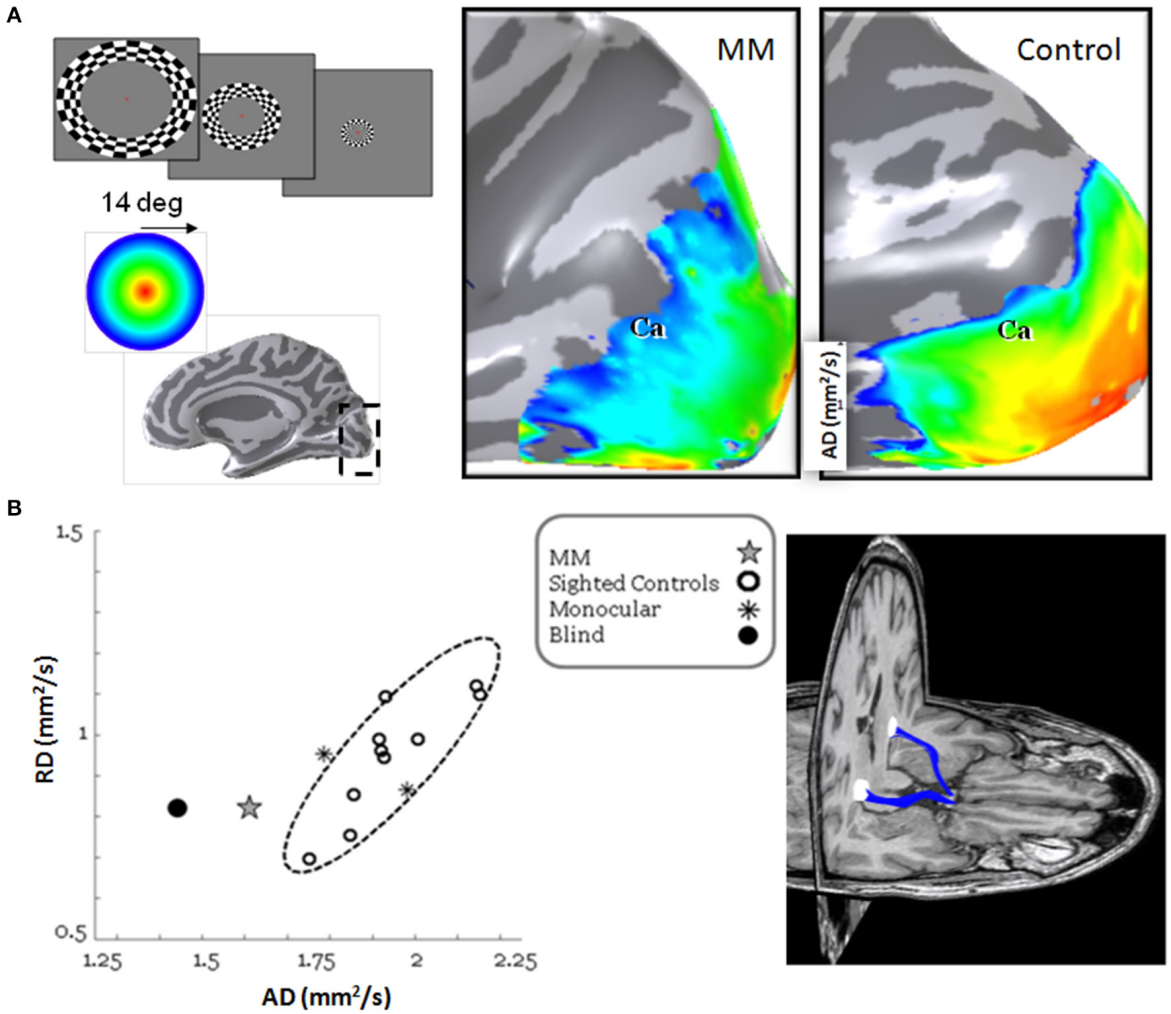
**Retinotopic maps and visual pathways reconstruction following long visual deprivation and restoration procedure. (A)** On the left: The expanding ring stimulus used to map the visual field eccentricity axis. Red represents mapping the center of the visual field, yellow the parafoveal region and blue- green the peripheral areas. On the right: Activation along the calcarine sulcus (Ca) overlaid on posterior medial view of the right hemisphere. In contrast to the normal eccentricity mapping where the center of the visual field has large representation in the occipital cortex, MM lacks this foveal representation. **(B)** On the right: three-dimensional rendering of the optic tract fibers (blue) shown superimposed on axial and coronal slices of MM's brain; note the missing left eye. The optic tracts connect the optic chiasm and the LGN (white sphere). On the left: scatter plot of the radial and axial diffusivities for the average of the right and left optic tracts. Data are from MM (gray star), 10 normal controls (black open circles), 2 seeing monocular subjects (black asterisks) and one blind subject (black closed circle). The 2-standard deviation covariance ellipsoid (dashed) is shown.

Functional MRI, including retinotopy mapping and population receptive field (pRF) estimation revealed several differences between MM and sighted controls (Levin et al., 2010); MM's retinotopic maps were almost entirely lacking the foveal representation. Furthermore, while posterior occipital regions typically include small pRFs, these cortical regions in MM were characterized by large pRFs. Using DTI and fiber tractography (Contrack algorithm Sherbondy et al., 2008), MM's optic tract and radiations were identified. While their anatomical appearance seemed intact, their microstructural measurements were significantly different from those of controls, with significantly impaired axial diffusivity (suggesting axonal loss) (see Figure 5).

MM's neuroimaging data suggest a specific cause of the limited recovery. His vision was interrupted at a time when visual neurons with small receptive fields were developing. Because these neurons are important inputs to the visual pathways of the brain, used for object recognition, MM has both poor resolution and limited ability to interpret objects.

This case serves as an example, which demonstrates that restoration of functional vision requires more than improving the retinal image contrast. To obtain functional vision, in addition to restoration of the sensing organ, the developmental trajectory of the individual and the consequences of the early deprivation on cortical circuitry need to be taken into account.

## Inflammatory damage to the optic nerve

Case 2: Optic neuritis is a demyelinating disease of the optic nerve, causing acute visual loss in young adults. Optic neuritis can be clinically isolated but more frequently appears as one of the manifestations of multiple sclerosis (MS). Early in the course of the disease the inflammatory process causes a significant reduction or even blockage in the amount of information being transferred along the optic nerve. This is expressed clinically as deficits in visual field, visual acuity, contrast sensitivity, and color perception. Later on, when the inflammation is alleviating, these functions are usually restored. Nevertheless, due to damage to the myelin surrounding the nerve fibers, there is a continuing delay in conduction along the optic nerve; which can last for several months or even years following the acute attack. Consequently, patients that are reported to have recovered according to standard visual testing continue to complain about residual visual dysfunction in their daily visual abilities. Recently, we demonstrated that static visual functions (visual acuity, visual fields, contrast sensitivity, and color perception) tend to recover shortly following the acute optic neuritis attack (weeks to months). Yet patients with optic neuritis continue to exhibit major deficiencies in dynamic visual functions (motion perception) even a year following the attack (Raz et al., 2011). We suggested that these perceptual differences between the ability to perceive static and dynamic visual functions reflect different visual processes; while static visual abilities depend solely on the amount of information being transferred along the optic nerve, the dynamic visual functions also depend on conduction velocity and therefore remain impaired for longer periods (Raz et al., 2012).

### The cortical reflection of motion perception deficit following optic neuritis

In recent years, fMRI has been used to examine the possibility that cortical mechanisms participate in the recovery process following peripheral visual insult, in addition to the afferent recovery processes. Previous fMRI studies on patients who had recovered clinically from optic neuritis showed an intact activation level in the object-related visual regions during stimulation of the affected eye. This was evident when activation in early visual areas was intact but also when it was reduced. Intact activation in higher visual areas was considered as evidence of cortical plasticity, where cortical adaptation to a persistent abnormal input contributes to the recovery process (Werring et al., 2000; Toosy et al., 2005; Levin et al., 2006; Korsholm et al., 2007; Jenkins et al., 2010).

**Figure 6:**
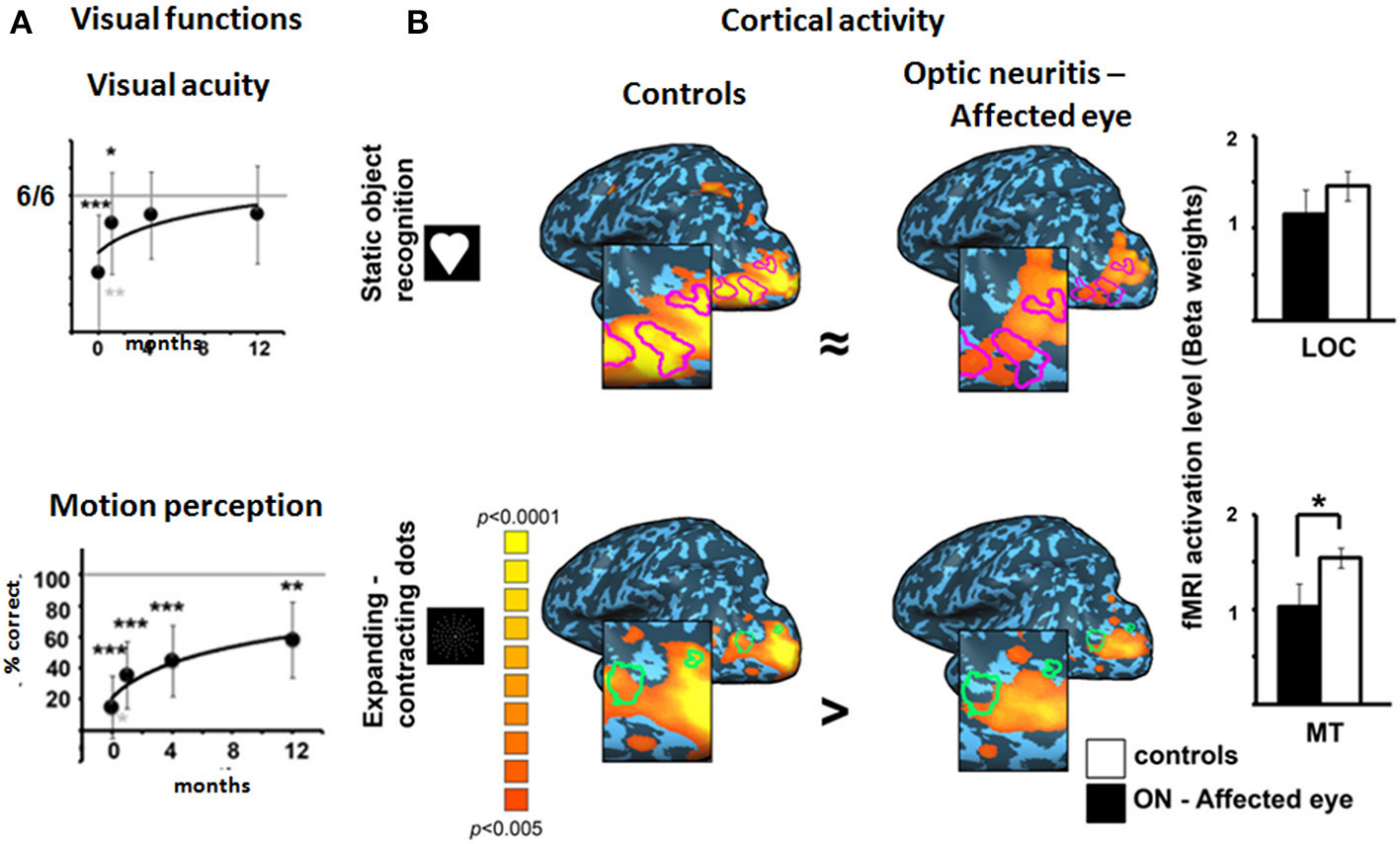
**Static and dynamic visual processing in optic neuritis patients: behavioral and cortical findings. (A)** Visual acuity (upper) and motion perception (expressed as the ability to extract motion-defined objects, lower panels) in the affected eyes of optic neuritis patients over time. (Patients' data is normalized to controls' mean). *N* = 21, 20, 18, and 14 at the acute, 1, 4, and 12 month time points, respectively. Black asterisks denote significant reduction in patients' values as compared to the controls. Gray asterisks denote significant change in patients' affected eyes' measurements between testing phases. ^*^p < 0.05; ^**^p < 0.01; ^***^p < 0.001. **(B)** fMRI activation maps showing activation within the object-related region (LOC) during static object recognition (upper) and within the motion-related region (MT) during motion processing (lower). The data are presented on a Talairach normalized inflated brain of the left hemisphere. LOC and MT are outlined on the lateral view of the cortex (LOC—purple lines, MT—green lines). Blow-ups highlight activation in the regions of interest (ROIs). Activation is seen for control subjects and affected eyes of optic neuritis patients. Color scale denotes significance levels. Bar graphs on the right denote the activation levels (beta weights) within each ROI for the two groups. Comparable cortical activation levels for the AEs and controls are found in LOC. However, a significant reduction in cortical activation during AE stimulation is evident in MT.

To assess the role of cortical plasticity in static and dynamic visual recovery, we compared cortical activity in response to these stimuli in patients 12 months following the optic neuritis attack (for further details on fMRI procedure see Raz et al., 2011). Figure 6 shows fMRI activation maps in optic neuritis patients and controls while viewing static objects and an expanding-contracting dots array. Activation is seen within the object and motion—related cortical regions (LOC and MT, respectively), which were first identified using separate functional localizers. While intact cortical activity was evident in visual areas responsible for object recognition, the activation in visual areas responsible for motion processing was reduced. Theses imaging results correspond to the behavioral data, reflecting the recovery of static but not dynamic visual processing 12 months following an optic neuritis attack.

We have proposed that the cortical activity following optic neuritis may reflect the visual percept (intact for visual acuity and impaired for motion perception) rather than demonstrating cortical plasticity, as suggested previously.

### The effect of demyelinative damage on neighboring white matter integrity

In recent years DTI and fiber tractography have been used to study the effect of focal damage to neighboring pathways. As was reported in animal studies, neuronal loss following damage is often greater than might be expected from the severity of the injury to the nerve itself. We utilized the visual pathways, which comprise a well-defined system, and optic neuritis, which is usually a spatially and temporally discrete event, to study the effect of focal demyelinative lesions on neighboring white matter.

**Figure 7:**
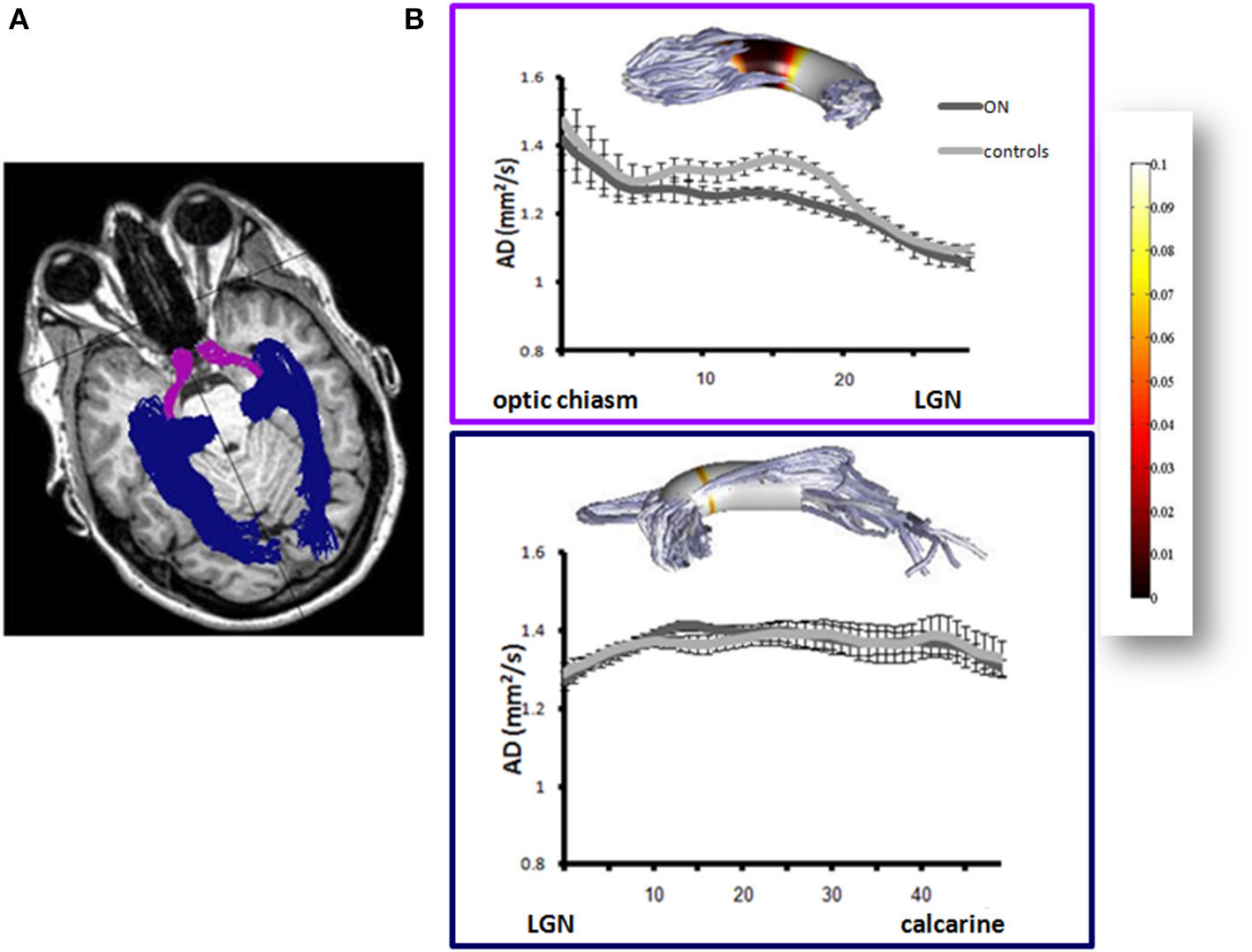
**Axial diffusivity in the optic tracts and radiations following optic neuritis. (A)** Fiber delineation: The optic tracts (purple) and optic radiations (blue) superimposed on T1 image in one control subject. **(B)** Axial diffusivity in the optic tracts (upper plots) and radiations (lower plots) of optic neuritis (*n* = 17) and control subjects (*n* = 12) (dark and light gray symbols, respectively). Diffusivities are presented at each point along the tract. Inserts above plots represent differences between ON and control groups at any point along the tract. Differences are represented as *p*-values (*T*-Tests, corrected for multiple comparisons). *P*-value scale is shown at the rightmost color-bar.

To assess this, we delineated the optic tracts and radiations in 17 optic neuritis patients (12–36 months following an optic neuritis attack) and 12 matched control subjects using DTI and fiber tractography methods and measured the directional diffusivities in those fibers. DTI data was acquired using a diffusion-weighted imaging sequence (2 mm thick slices covering the whole brain; *b* = 0 and *b* = 1000 s/mm^2^. The high *b*-value was obtained by applying gradients along 64 different diffusion directions). Image processing was done using the open-source mrVista package (http://vistalab.stanford.edu/software). Fiber tractography was performed using the probabilistic Contrack algorithm (Sherbondy et al., 2008). Optic tracts were estimated as the most likely pathways between the right or left sides of the chiasm, and the lateral geniculate nuclei (LGN) ROIs on the corresponding hemisphere. Optic radiations were estimated as the most likely pathways between the LGNs and the calcarine sulci ROIs in the corresponding hemisphere. Chiasm, LGN and calcarine ROIs were delineated for each subject on T1 images (Figure 7A). Diffusion measures along the optic tract and radiation bundles were re-sampled at 30 and 50 positions, respectively, calculating FA, AD, and RD at each of these nodes. In this way, measures throughout the length of the fiber could be combined across different subjects. In order to avoid partial voluming with non-white matter (i.e., ventricles or gray matter), diffusion measurements were taken near the dense core of the fibers.

Our results demonstrated reduced AD in the optic tracts of optic neuritis patients (Figure 7B). Furthermore, AD correlated with the corresponding Retinal Nerve Fiber Layer (RNFL) thickness in the patients' affected eyes (linear least-squares regression with calculation of the correlation coefficient *F* = 10.2; *p* = 0.01; *r* = 0.71). As opposed to the optic tracts, AD within the optic radiations was similar among patients and controls (Figure 7B). These findings suggest that axonal loss in the optic nerves of chronic optic neuritis patients proceeded to the optic tracts, demonstrating Wallerian degeneration. However, this process did not proceed to the optic radiations, and therefore did not support anterograde trans-neuronal degeneration. Despite the intact AD along the optic radiations, reduced FA and an elevated RD were evident in the patients (FA: 0.52 and 0.47 for controls and patients, respectively, *p* = 0.003 between group. RD: 0.58 and 0.62 for controls and patients, respectively, *p* = 0.046 between groups). Furthermore, FA and RD levels were associated with the presence of demyelinative lesions within the optic radiations (correlations between optic radiations intra-bundle lesions' volume and FA values: *F* = 5.8; *p* = 0.03; *r* = −0.53. Correlation between optic radiations intra-bundle lesions' volume and RD values: *F* = 10.7; *p* = 0.005; *r* = 0.65), suggesting that white matter damage in these fibers could be explained by local demyelinative damage in the optic radiations (Raz et al., 2014). Our results in the visual pathways of optic neuritis patients may model normal-appearing white matter (NAWM) pathology in MS, demonstrating that a demyelinative lesion in the proximal segment of the nerve fiber may result in chronic degeneration of its distal normal-appearing portion.

## Acquired chiasmatic insult

Acquired chiasmal abnormalities are usually categorized as intrinsic or extrinsic; involving the substance of the optic chiasm itself (e.g., in inflammatory processes), or resulting from a mechanical compression of adjacent structures (e.g., in adenomas) (Foroozan, 2003). Bitemporal hemianopsia is the classical visual-field-defect of disorders that involve the optic chiasm (Kirkham, 1972), due to the involvement of the crossing of nasal-retinal fibers of each optic nerve (leading to inability to view the temporal visual field of each eye). Resolution of inflammation or surgical removal of the compressing lesion can improve visual field and visual acuity.

### The cortical reflection of transient inflammatory bitemporal hemianopsia

**Figure 8:**
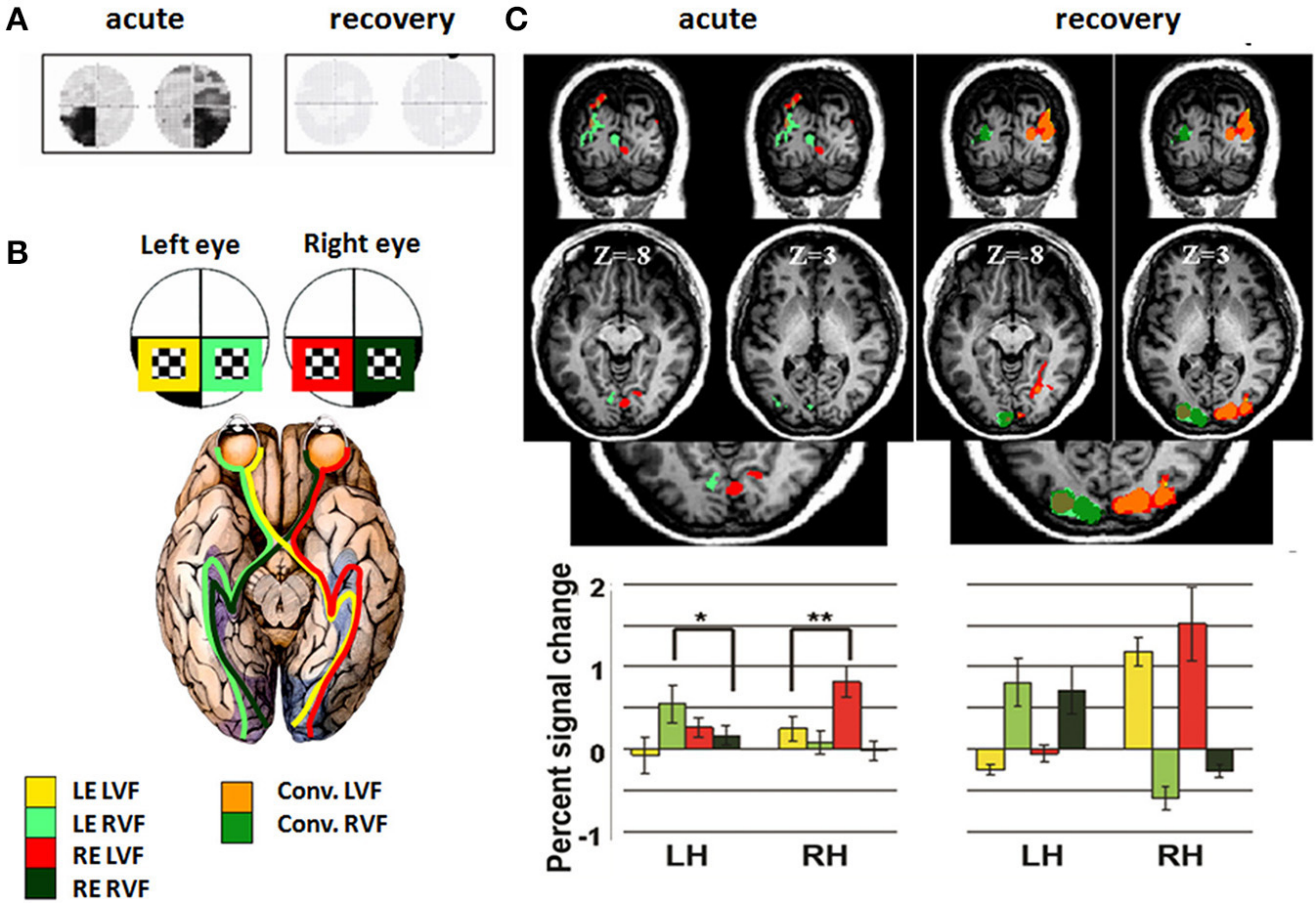
**Cortical activation patterns during acute chiasmal inflammation and following recovery. (A)** Patient's visual fields during the acute phase (left) and subsequent recovery (right). **(B)** fMRI study design: Four experimental conditions were used: (1) Stimuli projected to the LVF via the LE, marked in yellow; (2) Stimuli projected to the RVF via the LE, marked in light green; (3) Stimuli projected to the LVF via the RE, marked in red; (4) Stimuli projected to the RVF via the RE, marked in dark green. LVF—left visual field; RVF—right visual field; LE—left eye; RE—right eye. **(C)** Coronal and axial views of the fMRI activation patterns, elicited during the four experimental conditions. Activation is shown for the patient during acute phase (left) and following recovery (right). Same colors as in **(B)** (see scale). Conv. (convergence) refers to the overlap of activation elicited by stimuli projected to both eyes. Lower panel: Average activation level (percent signal change) during stimuli presentation in V1, showing the relative contribution of all experimental conditions. Asterisks denote significance level: ^*^p < 0.05; ^**^p < 0.01.

Case 3: We studied the effects of intrinsic optic chiasm damage in a 36-year-old woman, who presented with acute bilateral visual loss (Raz et al., 2010). Her past history included recurrent transverse myelitis episodes and a serologically positive test for Neuro-myelitis optica (NMO) antibodies. On admission, her visual fields demonstrated bitemporal defects, which were more pronounced in the lower visual field. T1, T2, and fluid attenuation inversion recovery–weighted MRI were performed in the acute phase. Several periventricular white matter lesions were evident, with no detectable optic chiasm abnormality. To further assess her bitemporal hemianopsia, an fMRI experiment was performed. During the fMRI scan, a flickering checkerboard was projected separately to each eye in each visual field. Stimuli were projected to the lower visual fields and were designed to fall within the scotoma of the patient when projected to her blind fields. This paradigm was carried out twice: during the acute phase and after visual recovery (8 months later). During the acute phase, visual cortical areas were activated only monocularly, whereas recovery was associated with the return of normal binocular input. These results confirm the association between bitemporal hemianopsia and an ipsilateral-only projection of the retinogeniculate pathway (Figure 8).

### The effect of chiasmal compressing meningioma on proceeding white matter integrity

**Figure 9:**
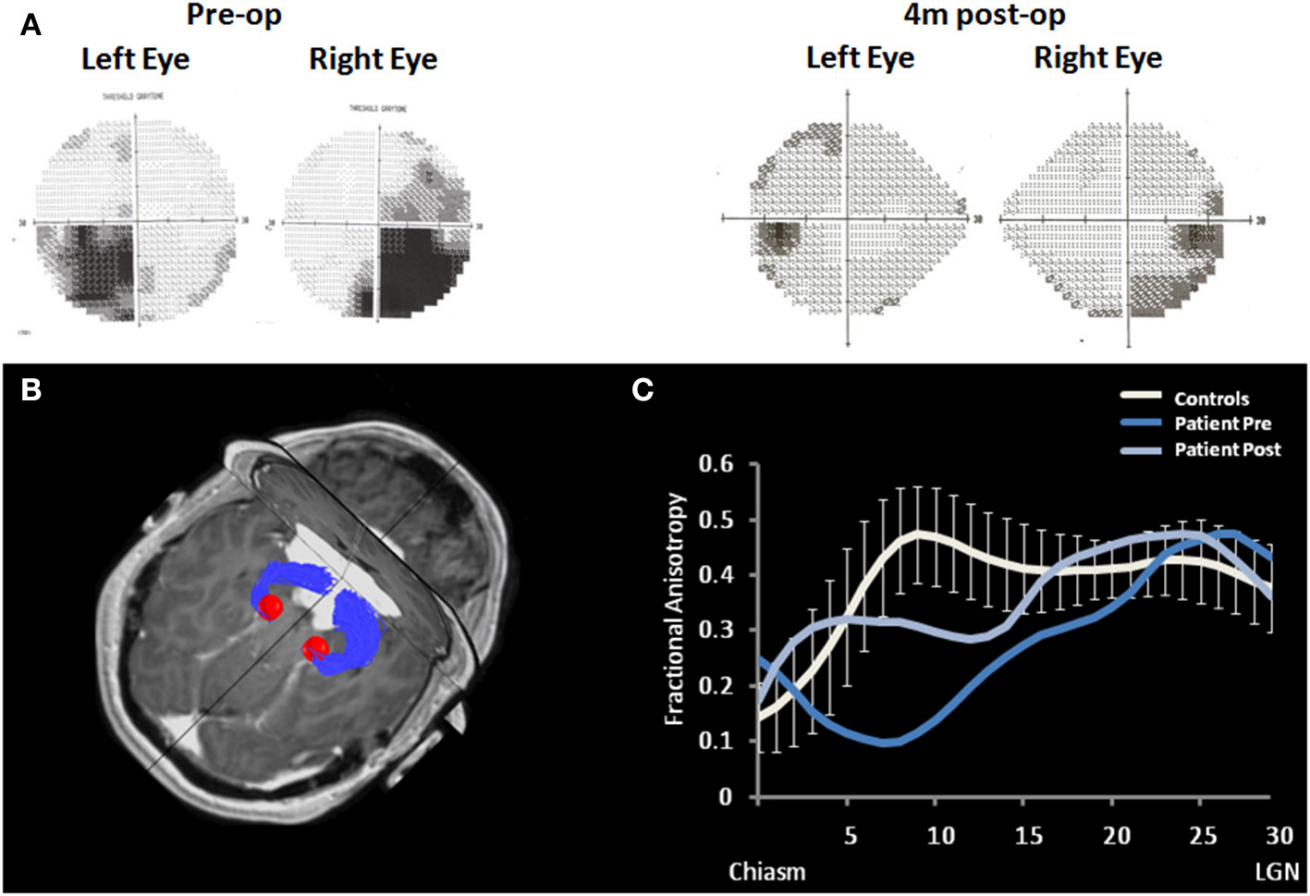
**Optic tracts' diffusivity measurement during chiasmal compression and following recovery. (A)** Patient's visual fields prior and following surgical removal of the meningioma. **(B)** Optic tracts delineation in the patient (blue fibers), superimposed on axial and coronal view. The compressing meningioma is seen in white. Red spheres represent the left and right LGNs. **(C)** Fractional anisotropy (FA) along the patient's optic tracts (averaged across the left and right sides) prior to and following surgical removal of the meningioma (dark and light blue, respectively). Patient's data is plotted against the mean FA of 17 age-matched control subjects (white plot). Error bars represent standard deviations. X axis defines position along the tract, from the chiasm to the LGN, given in arbitrary units, 30 positions were sampled in each subject.

Case 4: The effects of extrinsic optic chiasm damage were studied in a 35-year-old man presenting with bilateral hemianopsia, which he noticed 6 months prior to admission. Apart from the visual field loss, his ophthalmological examination was intact. Magnetic resonance imaging (MRI) showed a giant tuberculum sellae meningioma compressing the entire chiasm. The patient underwent right fronto-orbital craniotomy and the lesion was completely resected. Visual fields improved significantly, although some residual deficit was still evident (Figure 9A). To study the effect of the compression on the chiasm and optic tracts, DTI and fiber tractography (as detailed in case 2) were applied prior to and following surgical removal of the meningioma (Figure 9B). As seen in Figure 9C, the compression resulted in significant reduction of the FA as a measure of microstructure integrity (as compared to FA measured in the optic tracts of 17 age-matched control subjects). Following meningioma removal, FA was enhanced, although it remained significantly impaired. This sustained reduction in diffusivity may explain the residual visual loss following meningioma removal.

## Acquired visual cortical damage

### Functional mapping may explain a specific visual dysfunction

Case 5: A 65-year-old- woman was admitted to the rehabilitation department following diffuse occipital and occipito-parietal damage due to Posterior Reversible Encephalopathy Syndrome (PRES) (Hinchey et al., 1996). Behavioral tests suggested a specific deficit in face processing (inability to recognize famous faces despite the ability to recognize these persons following a verbal description, and a failure to recognize the pictures of her grandchildren). This deficit was seen, despite the intact processing of other object identity information (naming two-dimensional and three-dimensional visual objects, color naming). Such a behavioral deficit suggested specific damage in the brain areas responsible for face processing, while adjacent areas responsible for processing other objects remained intact. Since the distinction between damage in these different areas can only be demonstrated on the basis of function and in order to establish the specificity of face perception damage, the patient underwent an fMRI assessment (multi-slice gradient echo-planar imaging, 3 mm thickness axial slices covering the whole brain). Her examination included mapping of different areas along the hierarchy of the visual cortex, including primary visual areas and higher visual areas responsible for processing objects, motion, places, and faces. To map the object-related region (LOC), blocks of objects and scrambled version of these objects were contrasted; and to map motion-related regions (MT), blocks of expanding-contracting rings and stationary rings were contrasted. To compare activation for faces and places processing, blocks of famous faces, and places were shown. Data analysis was performed using the BrainVoyager QX software package (Brain Innovation).

**Figure 10:**
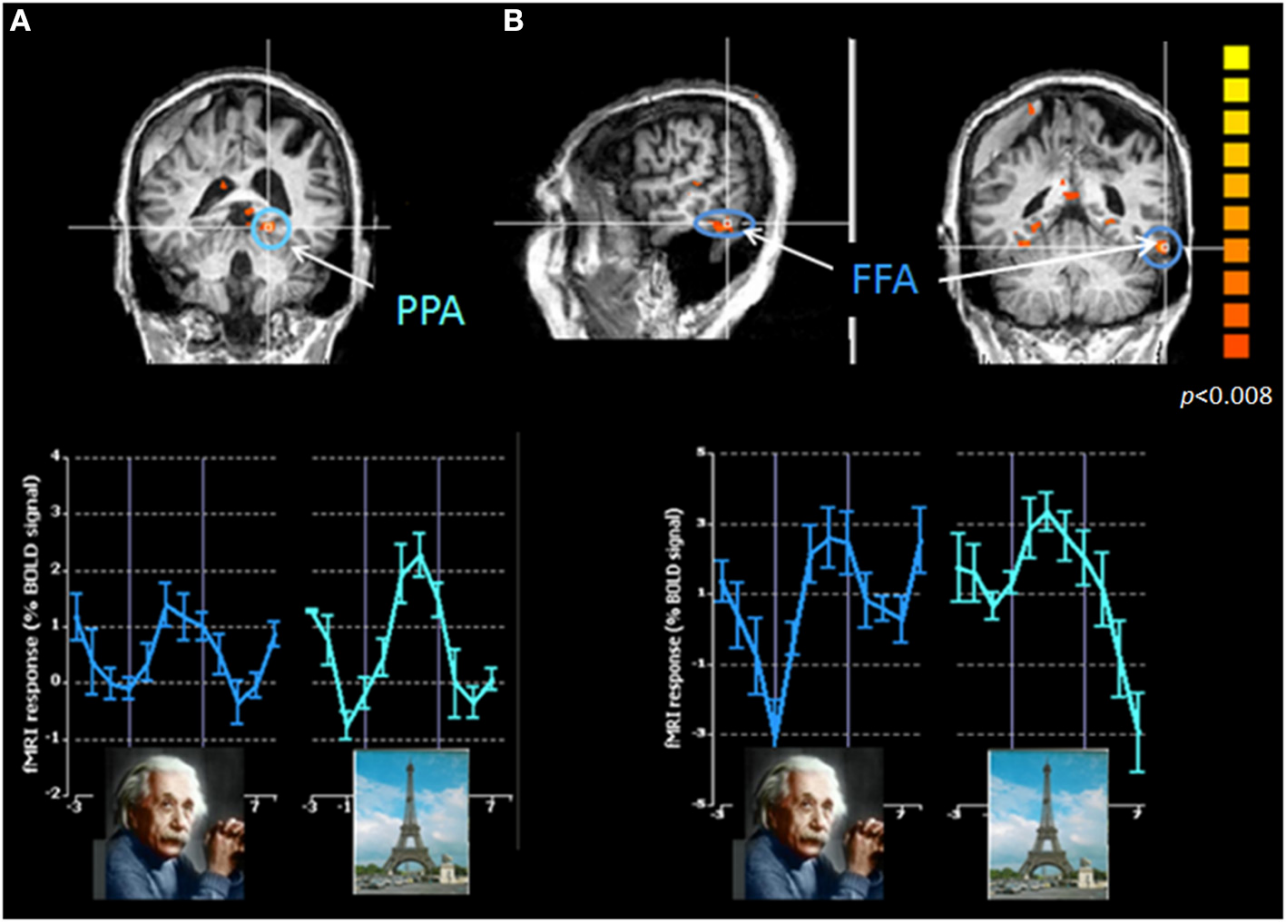
**Cortical activation patterns during faces and places recognition in a patient with acquired visual cortical damage: Correspondence between behavioral and cortical dysfunctions**. Cortical activation in the parahippocampal place area, PPA **(A)** and in the fusiform face area, FFA **(B)** during viewing pictures of faces and places. Upper panels represent the cortical activation maps centered on the two regions of interest (ROIs) (PPA marked in green, FFA marked in blue). Lower panels represent the activation elicited in each of these ROIs for the two stimuli type. Color scale denotes significance levels. The absence of cortical specification for processing faces corresponds to the patient's inability to process the identity of faces (while ability to process the identity of other visual stimuli is intact). MRI slices are shown in neurological convention.

The exam demonstrated a normal pattern of activation in early visual areas, as well as in areas responsible for objects and motion processing. In the parahippocampal place area (PPA), which is known to be involved in processing topographical scenes, greater activity was evident when viewing pictures of places as compared with pictures of faces (a pattern that resembles normal-sighted subjects). In contrast, no specification for face recognition was evident in the ventral-temporo-occipital fusiform face area (FFA), responsible for face processing (Figure 10). PPA and FFA were defined anatomically. This case clearly demonstrates the ability of functional imaging to bridge the gap between the clinical deficit and the associated cortical activity. The fMRI examination revealed an unusual pattern of activation in an area known to be responsible for face recognition, which anatomically appeared intact (via standard MRI regime).

### Functional mapping may predict recovery of visual dysfunction

Case 6: A 64-year-old woman was admitted to our clinic following repeated occipital ischemic events. She reported no light perception and behaved as though she was blind. Standard MRI revealed damage in her right early visual regions. Flash visual evoked potential (VEP) testing demonstrated delayed but evident cortical responses. To reveal whether the visual cortex received and processed the visual input, an fMRI examination was performed. fMRI examination included viewing full-field flickering checkerboard to activate early visual regions; viewing visual objects (vs. scrambled images of these objects) to activate LOC; viewing pictures of faces (vs. scrambled images of these faces) to activate FFA; and viewing expanding-contracting rings (vs. stationary rings) to activate MT. In addition, resting-state fMRI was applied to assess the visual cortical network.

Viewing flickering checkerboard resulted in intact activation at early visual regions within the undamaged left hemisphere. Intact activation was also evident within higher-order visual regions: activation of LOC in response to viewing objects, activation of the FFA in response to viewing faces, and activation of MT for viewing moving stimuli.

**Figure 11:**
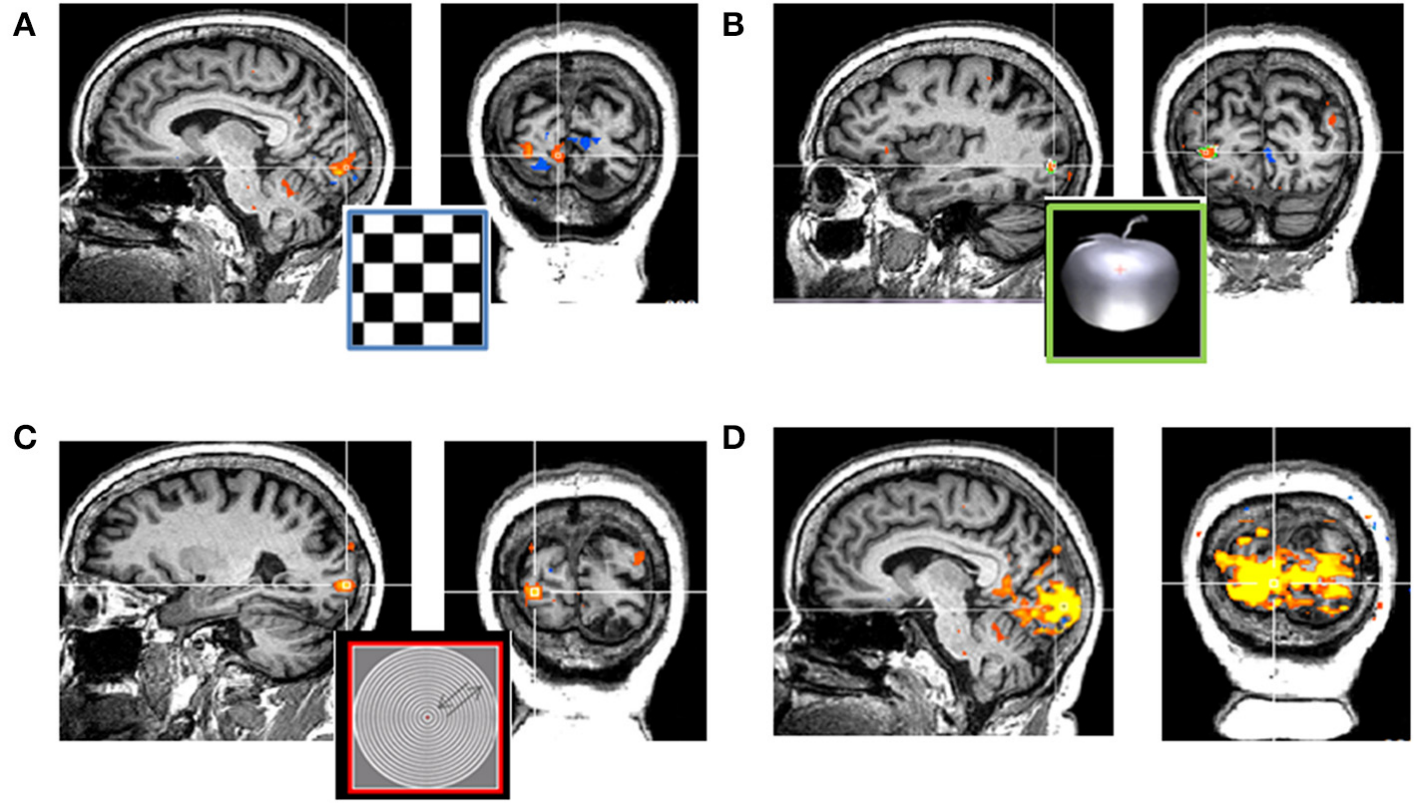
**Cortical activation patterns during visual processing in a patient with acquired visual cortical damage: No correspondence between behavioral and cortical dysfunctions. (A)** Cortical activation in primary visual cortex during viewing flickering checkerboard stimuli. **(B)** Cortical activation in the object related region—LOC during viewing pictures of objects (as compared to viewing their scramble version). The orange plus on the apple represents fixation cross. **(C)** Cortical activation in the motion related region—MT during viewing moving stimuli (as compared to viewing static stimuli). **(D)** Cortical activation during resting-state fMRI (while no visual stimulus is presented). The visual cortical network is shown, extended over a robust part of the posterior occipital cortex and among the two hemispheres. Yellow to orange clusters represent positive activation, blue clusters represent deactivated cortical regions. These intact cortical activation patterns contradict the patient's reported inability to perceive light. MRI slices are shown in neurological convention.

Functional connectivity during resting-state fMRI revealed an intact visual cortical network (Figure 11), accompanying early and higher-order visual regions. The sum of these fMRI results suggest intact processing of visual information within the visual cortical regions. Thus, the absence of light perception may result from unawareness of visual processing rather than dysfunction of the visual cortical regions. Demonstration of the intactness of visual processing is a prerequisite step for rehabilitation.

## Conclusions

Vision is a perceptual experience, which includes the retinal ability to perceive inputs as well as the cortical ability to process them. Vision is a complicated process of sorting, encoding, interpreting, and understanding the meaning of the visual input. Consequently, the image created in our awareness does not always reflect the optical reality. Areas devoted to visual information processing constitute a large portion of the human cortex, indicating their complexity. The visual cortex contains an impressively large number of distinct areas and specific functional networks that create the neuronal basis for perceiving different visual elements of the stimuli (Van Essen and Drury, 1997). These specifications in visual processing are manifested via clinical cases, for example the inability to recognize faces while other visual abilities are intact.

In the past two decades, the use of non-invasive functional imaging and especially fMRI has dramatically expanded our knowledge about the functional visual human brain in healthy and disease states. Intact vision requires functioning of the entire visual system, starting at the eye, continuing through the visual pathways and ending at the visual cortex. Furthermore, the visual cortex is not isolated, and functioning separately from other areas won't achieve normal vision. Therefore, we should relate to the visual system with a holistic view, integrating imaging techniques for the eye, the visual pathways and the brain, in order to assess its overall functioning.

Taking advantage of their ability to visualize the whole brain at once, DTI and fMRI are adequate tools to assess the consequences of focal lesions along the visual pathways on white matter integrity and cortical functioning in the entire system.

As in the case of optic neuritis, DTI is useful for investigating the effect of focal lesions on neighboring white matter, modeling the relationships between intact and pathological white matter structures, or, as discussed in the case of chiasmal compression, DTI may explain residual visual loss after removal of the focal lesion.

Evaluating the functional properties of the entire brain, fMRI is useful for differentiating between functioning and dysfunctioning cortical regions, and explaining specific visual dysfunctions, as was seen in the case of acquired brain damage.

Regarding the system as a whole is important when developing advanced technologies to reconstruct the sensing organ. As the first case illustrates, cutting edge studies aimed at restoring vision such as retinal prosthesis or retinal stem cell transplant, cannot only focus on reconstruction of the sensing organ. They also have to account for the intactness or rehabilitation of the entire system.

fMRI can bridge the gap between MRI-detected tissue damage and clinical manifestations, explaining behavioral phenomena even in the absence of a clear structural fingerprint (Filippi and Rocca, 2003).

In the optic neuritis studies we emphasized the association between cortical activity and visual functioning, demonstrating motion perception deficits in both. In this way, the cortical activity reflects the behavioral phenomenon. In contrast, the last case demonstrated a mismatch between the behavior and the cortical activity pattern. Intact activation within the occipital visual cortex was evident despite the patient experiencing no light perception. These findings were used to leverage the rehabilitative process since the cortical residua indicated possible recovery.

The examples reviewed above emphasize the importance of using functional imaging to understand visual system pathologies. In some cases, standard MRI scans do not have enough resolution to localize the specific pathology. The clinical picture cannot be explained by clear structural damage and an understanding of the pattern of cortical activity and connectivity between different cortical and subcortical areas is needed to explain the visual deficit. To that end we recommend using advanced imaging techniques to better understand the neuronal basis of the apparent neurological event.

### Conflict of interest statement

The authors declare that the research was conducted in the absence of any commercial or financial relationships that could be construed as a potential conflict of interest.
